# Use of Adjuvant Sorafenib in Liver Transplant Recipients with High-Risk Hepatocellular Carcinoma

**DOI:** 10.1155/2014/913634

**Published:** 2014-04-10

**Authors:** Kirti Shetty, Chiranjeev Dash, Jacqueline Laurin

**Affiliations:** ^1^Johns Hopkins Medicine, Division of Gastroenterology and Hepatology, Sibley Memorial Hospital, 5215 Loughboro Road, NW Suite No. 300, Washington, DC 20016, USA; ^2^Georgetown University School of Medicine, Lombardi Cancer Center, 3800 Reservoir Road NW, Washington, DC 20007, USA; ^3^Georgetown University Hospital, Georgetown Transplant Institute, 3800 Reservoir Road NW, Washington, DC 20007, USA

## Abstract

The efficacy of liver transplantation (LT) for hepatocellular (HCC) is limited by tumor recurrence rates of 10–15%. We undertook this pilot study to examine the use of sorafenib as adjuvant therapy in high-risk LT recipients. *Methods*. We prospectively enrolled patients transplanted for HCC into a treatment protocol utilizing sorafenib if their explant examination showed evidence of viable tumor exceeding Milan criteria. We utilized as historical controls patients transplanted previously, whose explant tumor characteristics exceeded Milan criteria, but who were not “preemptively” treated with sorafenib. Wilcoxon two-sample test and Fisher's exact test were used to compare survival and recurrence rates between the two groups. *Results*. Seven patients were treated with sorafenib and compared to 12 historical “controls.” Two of 7 treated patients suffered from HCC recurrence. Of the comparison group, 9 experienced HCC recurrence and all succumbed to disease. Dose reduction improved tolerance of drug. The overall rate of HCC recurrence was decreased in the adjuvant therapy group compared to historical controls (29% versus 75%, *P* = 0.07). Disease free 1-year survival for the treated versus untreated group was 100% versus 66%, respectively. *Conclusion*. Adjuvant use of sorafenib is safe and decreases risk of HCC recurrence in high-risk LT recipients.

## 1. Introduction

Hepatocellular carcinoma (HCC) accounts for more than a million new cases each year worldwide and is the third leading cause of cancer-related death worldwide. Its incidence in the United States has shown a dramatic rise over the past few decades [1] and is expected to increase in the coming years.

Liver transplantation (LT) is in many cases the ideal therapy for HCC, providing not only oncologic resection but also replacement of a diseased organ. An important study by Mazzaferro and colleagues demonstrated that if LT was limited to those with early HCC, long-term post-LT survival was excellent [2]. Based on this study and others, LT has become the standard of care for those HCC that satisfy the eponymous Milan criteria (a single tumor under 5 cm, or ≤3 tumors each under 3 cm, without evidence of metastatic disease/vascular invasion) on radiological imaging. However, despite advances in imaging techniques, almost 20% of HCCs are “understaged” on pre-LT radiological studies [3] and are found on explant to exceed Milan criteria, an established risk factor for post-LT HCC recurrence. Specific predictors of post-LT recurrence are tumor size, multifocality, and vascular invasion. The efficacy of liver transplantation for HCC is therefore limited by tumor recurrence and the lack of effective preventive strategies for those at high recurrence risk.

Sorafenib is a multiple tyrosine kinase inhibitor, which inhibits tumor angiogenesis by inhibition of the vascular endothelial growth factor (VEGF) and platelet derived growth factor (PDGF) signaling pathway and is the only FDA-approved systemic chemotherapeutic agent for the treatment of advanced HCC at this time [4, 5]. We therefore undertook this pilot study to examine the preemptive use of sorafenib in the prevention of HCC recurrence post-LT in those with high-risk explant characteristics. We sought to assess the tolerability and safety of sorafenib in this population and examined rates of HCC recurrence and post-LT survival.

## 2. Methods

Patient undergoing LT for HCC after 1/1/08 were considered for entry into the study. All explants were sectioned at 5 mm intervals and examined by an experienced hepatopathologist. Patients were offered entry if their explant examination demonstrated viable tumor exceeding Milan criteria, specifically any of the following.

(1) 3 or more viable tumors (if 3 tumors, at least one over 3 cm diameter), (2) one viable tumor greater than 5 cm in diameter, or (3) evidence of micro- or macrovascular invasion.

Exclusion criteria were as follows: pathologic features of mixed hepatocellular cancer with cholangiocarcinoma, contraindication to sorafenib, absence of informed consent, and use of sirolimus as part of the immunosuppressant regimen. The study was approved by our Institutional Review Board. Patients were started on sorafenib 200 mg by mouth once daily within 12–24 weeks of LT, and the dose was titrated upwards to the target dose of 400 mg twice daily. Adverse reactions were monitored using the National Comprehensive Cancer Network (NCCN) guidelines. Sorafenib doses were adjusted according to the side effects reported by the patients. Patients underwent abdominal MRI and CT scan of the chest every three months or earlier if clinically indicated, for HCC surveillance. The 7 patients who satisfied entry criteria were maintained on standard immunosuppression, and sirolimus use was prohibited.

To identify a comparison group, we reviewed explant pathology of all patients transplanted for HCC from 2003 (initiation of the liver transplant program at our institution) onwards. We identified 12 patients who exceeded Milan criteria on explant examination and who did not receive sorafenib preemptively after LT. Of these, 9 patients had undergone LT prior to protocol initiation, and 3 patients declined treatment with sorafenib when offered entry into this protocol. There were no significant changes in the immunosuppression protocols or the pretransplant treatment of HCC between the two groups.

The primary endpoints were toxicity and tolerability of sorafenib. Secondary end points included rate of HCC recurrence, time to recurrence, and mortality. All patients were planned for one year of treatment with sorafenib. Demographic characteristics, clinical variables, and transplant outcomes of patients included in the study were compared using means for continuous variables and frequencies for categorical variables. Differences in characteristics between the adjuvant therapy group and historical comparison group were compared using the Fisher's exact test and *t*-test. Posttransplant survival and HCC recurrence between the two groups were compared using Kaplan-Meier (K-M) curves.

## 3. Results

### 3.1. Demographic Data

Baseline characteristics are summarized in Table 1. The majority of patients in both groups were male, and hepatitis C virus (HCV) represented the primary underlying etiology of liver disease.

### 3.2. Pretransplant Imaging and Explant Tumor Characteristics

All patients underwent either contrast-enhanced magnetic resonance imaging (MRI) or computerized tomographic (CT) scan prior to LT. Imaging and explant characteristics are summarized in Table 2. Of note, even though no patients were noted to have >3 nodules on pre-LT imaging, 50% and 42.8%, respectively, in the historical and the treated group were found to have multifocal disease on explant examination. Vascular invasion was noted on microscopic examination of the explants (i.e., microvascular invasion) in 33% and 28% of patients in each group. This was, by definition, not detectable on pretransplant imaging.

The mean alpha-fetoprotein (AFP) level at LT was not significantly different between groups. All patients received locoregional therapy (LRT) prior to LT, except 1 patient in the treatment group, with an equivalent number of LRT sessions between groups. Five patients with recurrent HCC in the historical comparison group were treated with sorafenib after recurrence was documented.

There were no significant differences between the two groups in terms of the number of tumors, presence of microvascular invasion, mean/maximum tumor diameter, tumor differentiation, and percentage of necrosis.

### 3.3. Side Effects

The most frequent side effects of sorafenib were skin rash and diarrhea (58.3% of treated patients experiencing either or both adverse effects). Twenty-two percent of patients experienced “hand-foot” syndrome. Nausea and fatigue were also noted as side effects of sorafenib. There were no differences in the adverse effect profile of patients treated “preemptively” versus for recurrent disease.

For the first 3 patients, dose reduction was required to achieve tolerance. Thereafter, patients were started at low doses of 200 mg daily and increased to full doses (400 mg twice daily) as tolerated.

### 3.4. HCC Recurrence and Survival

The mean follow-up for treated patients was longer than that for those in the historical group but this difference did not reach statistical significance (1125 versus 840 days, *P* value = 0.18).

The overall rate of HCC recurrence was lower in the adjuvant therapy group compared to historical controls, (29% versus 75%, *P* = 0.07, Table 3). Disease-free 1-year survival for sorafenib and the comparison group was 100% and 66%, respectively; overall 1-year survival was 100% and 92%, respectively. Two of 7 sorafenib treated patients suffered HCC recurrence (at 14 months and 52 months), one patient is deceased; the other is alive 5 months following documented recurrence. Of the comparison group, 9 out of 12 patients suffered HCC recurrence, and all succumbed to the disease. There were no alternate causes of death in either group. All recurrences were biopsy proven. The median time to recurrence among those treated with adjuvant therapy was longer but not statistically significant (1053 days versus 620 days, *P* = 0.08).

The lung represented the most common site of recurrence (6 patients), followed by the liver (3 patients); 2 patients had multiple sites of recurrence. Survival rates at study conclusion were higher for the treatment group (85.7%) compared to the historical arm (33.3%) One patient is alive at the current time 4 months from date of documented recurrence.

**Figure 1:**
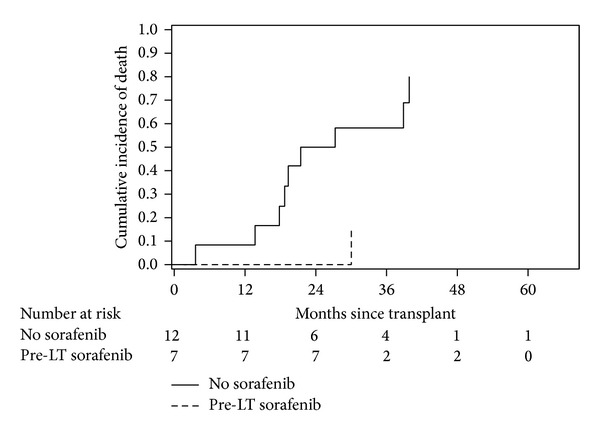
Mortality transplant patients with high-risk hepatocellular carcinoma at the Goergetown Transplant Institute, Washington, DC, by use of adjuvant pre-LT sorafenib.

The mortality incidence for the two groups is presented in Figure 1. It can be seen that the historical comparison group appears to have a higher mortality (75%) compared to the adjuvant therapy group (14%). However, because of the small sample size and the large number of censored observation, the sample size was effectively only 9 patients. This number is too small to use the tests of homogeneity over strata that require larger sample sizes in order to be meaningful.

## 4. Discussion

LT offers excellent survival outcomes in patients with HCC who satisfy well-established selection criteria. These criteria are however based on pre-LT imaging studies, which have been shown to underestimate tumor stage in approximately 20% of patients. Patients with explant characteristics of tumor multifocality and vascular invasion are at high risk of recurrence. We have previously shown [3] that, in those who exceed Milan criteria on explant, the hazard ratio for HCC recurrence increased to 3.14 (*P* value 0.03) compared to those whose HCCs on explant satisfied Milan criteria.

As a consequence, HCC recurrence after LT occurs at a significant rate, ranging from 3.5 to 26%, with median rates of 13%. This represents an important limitation to the efficacy of LT for malignant liver disease and may not be an appropriate use of an increasingly scarce resource. There are no established preventive strategies for HCC recurrence after LT. Adjuvant chemotherapy with 5-fluorouracil/carboplatin, cisplatin/adriamycin, or doxorubicin has been studied, but published reports document conflicting efficacy rates and an unfavorable toxicity profile [6, 7].

The use of conventional chemotherapeutic regimes appears to be constrained by toxicity and difficulty of administration. The recent development of molecularly targeted therapies with acceptable side effect profiles and oral availability may provide an opportunity to reevaluate our approach to adjuvant treatments. Sorafenib is the first of these agents to have been FDA approved for the treatment of advanced HCC [5], and we believe it offers an opportunity for use in the prevention of HCC recurrence in high-risk individuals following LT.

Two recent publications address the utility of sorafenib in a post-LT population. Saab et al. [8] described 8 patients treated with adjuvant sorafenib therapy and reported recurrence rates of 12.5%, as compared to 50% in a control population. As in our population, sorafenib appeared safe. A study from China consisted of a somewhat ill-defined population [9], but also appeared to confirm overall enhanced survival rates in patients offered sorafenib. Our study comprises a homogenous group of patients, carefully monitored for recurrence with a longer period of follow-up than previously reported. As a result, we are able to report on delayed recurrence (52 months) in one of the treated patients. While it is not possible to base mechanistic insights on such a limited sample, our experience raises tantalizing questions about the mechanism of action of sorafenib suggesting that it may render tumor cells “dormant” rather than destroyed.

Studies that have examined the use of sorafenib prior to liver transplantation have shown mixed results, with a suggestion of increased biliary complications and acute cellular rejection following LT [10, 11]. Several studies have examined sorafenib as treatment for recurrent HCC following LT [12–14]. These studies have documented a similar side effect profile as noted in our study. Other therapies have included surgery, systemic chemotherapy, as well as mammalian target of rapamycin (m-TOR) inhibitors [14, 15]. Overall results have been disappointing, suggesting that recurrent HCC following LT is invariably a lethal and unresponsive disease.

Our small pilot study was primarily designed to assess the safety and tolerability of sorafenib. We found that sorafenib was well tolerated in the majority of patients, with increased tolerability if the dose was gradually titrated upward instead of started at a high dose. The main adverse event was a skin rash, which usually responded well to topical therapy and dose adjustment. None of the patients required permanent dose discontinuation. There were no observed interactions with immunosuppressant regimens and no episodes of acute cellular rejection. This study was not sufficiently powered to detect differences in overall survival rates. However, there was a striking reduction in the rate of recurrence among treated, as compared to a historical comparison group, matched by explant features. All but one patient with HCC recurrence have succumbed to their disease, and hence the overall mortality rate in the historical control group was much higher (75%) compared to treated patients (14%). Both preemptively treated patients who developed recurrence did so at a much later time point than those who were not treated, raising the possibility that sorafenib may delay the time to recurrence. However, the 5 treated patients without recurrence have now been followed for a mean time of 1125 days and there was no statistically significant difference in follow-up times. Our study suffers from the limitation of being a single-center experience with a small number of patients. The comparison group was historical and therefore could not be controlled for many variables.

In summary, we have demonstrated that the use of sorafenib in patients transplanted for high-risk HCC is a safe, feasible, and well-tolerated intervention. In our small sample, sorafenib use was significantly associated with a lower risk of HCC recurrence and improved survival. Larger, prospective controlled trials will be required and have in fact been planned, to further examine the preemptive use of sorafenib and similar agents in this population, so that we may expand the armamentarium of therapies that are available to this challenging group of patients.

## Figures and Tables

**Table 1:** Demographic characteristics.

	Sorafenib adjuvant therapy *n* = 7	Historical comparison group, *n* = 12
Average age ± SD (years)	56.1 ± 10.5	56.7 ± 6.0
Male (%)	6 (85.7)	10 (83.3)
HCV (%)	6 (85.7)	9 (75.0)
Mean HCC size ± SD (mm)	39.00 ± 21.21	37.50 ± 15.86
Mean AFP at OLT ± SD (ng/mL)	794 ± 1229	880 ± 1278
Mean lowest AFP after OLT ± SD (ng/mL)	3.96 ± 5.30	65.35 ± 204.02
Mean number of TACE* treatments on each patient	1.71	2.16

*P* values between groups not significant and not reported.

*Transarterial chemoembolization.

**Table 2:** Pre-LT imaging and explant tumor characteristics.

	Sorafenib adjuvant therapy *n* = 7		Historical comparison group *n* = 12	
	Imaging	Explant	Imaging	Explant
Tumor *N* > 3	0	3 (42.8%)	0	6 (50%)
Maximum tumor size (cm)	5.5	8	8	7
Mean tumor diameter (cm)	2.86 (1.34)	3.90 (2.12)	3.38 (1.84)	3.75 (1.59)
Vascular invasion	0	2 (28.5%)	0	4 (33%)
Histological grade**				
Well/moderate		3		4
Poor		4		8

*P* values between groups not significant and not reported.

**Modified Edmondson criteria [16].

**Table 3:** HCC recurrence and survival outcomes.

Response	Sorafenib adjuvant therapy *n* = 7	Historical comparison group *n* = 12	*P* value
Recurrent HCC (%)	2 (29)	9 (75)	0.07
Median recurrent time from OLT (days)	1053	620	0.08
1-year disease-free survival	7 (100)	8 (66)	
1-year overall survival (%)	7 (100)	11 (91.6)	0.63
Mean follow-up duration in days (SD)	1125 (310)	840 (483)	0.18

## Abbreviations

HCC: Hepatocellular carcinoma
LT: Liver transplantation
VEGF: Vascular endothelial growth factor
PDGF: Platelet derived growth factor
NCCN: National Comprehensive Cancer Network
K-M: Kaplan-Meier
HCV: Hepatitis C virus
MRI: Magnetic resonance imaging
CT: Computerized tomographic
AFP: Alpha-fetoprotein
LRT: Locoregional therapy.
